# Bonferroni-Holm and permutation tests to compare health data: methodological and applicative issues

**DOI:** 10.1186/s12874-018-0540-8

**Published:** 2018-07-20

**Authors:** Massimiliano Giacalone, Zirilli Agata, Paolo Carmelo Cozzucoli, Angela Alibrandi

**Affiliations:** 1 Department of Economics and Statistics, University of Naples Federico II, 80126 Naples, Italy; 2 Department of Economics, Unit of Statistical and Mathematical Sciences, University of Messina, 98100 Messina, Italy; 3 Department of Economics, Statistics and Finance, University of Calabria, 87036 Rende Cosenza, Italy

**Keywords:** Permutation tests, Bonferroni-Holm procedure, Multiplicity control, Inflammatory bowel diseases, Comparative analysis

## Abstract

**Background:**

Statistical methodology is a powerful tool in the health research; however, there is wide accord that statistical methodologies are not usually used properly. In particular when multiple comparisons are needed, it is necessary to check the rate of false positive results and the potential inflation of type I errors. In this case, permutation testing methods are useful to check the simultaneous significance level and identify the most significant factors.

**Methods:**

In this paper an application of permutation tests, in the medical context of Inflammatory Bowel Diseases, is performed. The main goal is to assess the existence of significant differences between Crohn’s Disease (CD) and Ulcerative Colitis (UC). The Sequentially Rejective Multiple Test (Bonferroni-Holm procedure) is used to find which of the partial tests are effectively significant and solve the problem of the multiplicity control.

**Results:**

Applying Non-Parametric Combination (NPC) Test for partial and combined tests we conclude that Crohn’s Disease patients and Ulcerative Colitis patients differ between them for most examined variables. UC patients compared with the CD patients, have a higher diagnosis age, not show smoking status, proportion of patients treated with immunosuppressants or with biological drugs is lower than the CD patients, even if the duration of such therapies is longer. CD patients have a higher rate of re-hospitalization. Diabetes is more present in the sub-population of UC patients. Analyzing the Charlson score we can highlight that UC patients have a more severe clinical situation than CD patients. Finally, CD patients are more frequently subject to surgery compared to UC. Appling of the Bonferroni Holm procedure, which provided adjusted *p*-values, we note that only nine of the examined variables are statistically significant: Smoking habit, Immunosuppressive therapy, Surgery, Biological Drug, Diabetes, Adverse Events, Re-hospitalization, Gender and Duration of Immunosoppressive Therapy. Therefore, we can conclude that these are the specific variables that can discriminate effectively the Crohn’s Disease and Ulcerative Colitis groups.

**Conclusions:**

We identified significant variables that discriminate the two groups, satisfying the multiplicity problem, in fact we can affirm that Smoking habit, Immunosuppressive therapy, Surgery, Biological Drug, Diabetes, Adverse Events, Hospitalization, Gender and Duration of Immunosoppressive Therapy are the effectively significant variables.

## Background

Statistical methodology is an useful and powerful tool in the medical scientific research; therefore, an important increase in the use of statistical methods having been documented in most medical journals [23, 32, 46].

In recent years permutation tests increased in applications to solve complex multivariate problems. Permutation tests are essentially of an exact nonparametric nature in a conditional context, where conditioning is on the pooled observed data set which is often a set of sufficient statistics in the null hypothesis. Whereas, the reference null distribution of most parametric tests is only known asymptotically [39].

There are, however, many complex multivariate problems (quite common in biostatistics, clinical trials, engineering, the environment, epidemiology, experimental data, industrial statistics, pharmacology, psychology, social sciences, etc.) that are difficult to solve outside the conditional framework and in particular outside the method of Non Parametric Combination (NPC) of dependent permutation tests [38].

Permutation tests and bootstrap methods have very wide-ranging applications, both share a common potential drawback: as data-intensive resampling methods, both can be runtime prohibitive when applied to large or even medium-sized datasets. The data explosion over the past few decades has made this a common occurrence and it highlights the increasing need for faster and more efficient permutation tests and bootstrap algorithms [31]. The permutation test essentially works by combining two important principles: exchangeability and conditioning.

The main goal of this paper is, applying the NPC test methodology, to study a specific medical problem with a large amount of patients (about 1700) in order to assess the existence of significant differences between subjects affected by two Inflammatory Bowel Diseases (IBD); in particular, Crohn’s Disease (CD) and Ulcerative Colitis (UC), with reference to a great number of variables. In this case we are in presence of an authentic real complex problem to be solved; for its solution, the permutation methods are better than the ordinary parametric methods because do not require strong assumptions that are extremely difficult to justify. Since several variables are considered, we also propose an application of the Bonferroni-Holm procedure for the multiplicity control. In the paper theoretical, methodological and applied aspects [44] have been fruitfully integrated with specific competences from medicine field [33].

### The medical context: IBD

The inflammatory bowel diseases (IBD) are chronic inflammatory diseases of the intestinal mucosa; they include only Crohn’s Disease (CD) and Ulcerative Colitis (UC).

- Crohn’s Disease can affect the entire gastrointestinal tract, from mouth to anus. In about 90% of cases, the disease mostly affects the last part of the small intestine (ileum) and the colon. It is characterized by intestinal ulcers, often alternating with stretches of healthy gut, which, if not properly treated, can lead to complications (such as stenosis or fistula) that may require surgery. Immunosuppressive therapy and regular monitoring are used to control the disease and its progression in most cases.
- Ulcerative Colitis primarily affects the rectum and may involve part or all of the colon. The main clinical symptoms are diarrhoea, often with blood and mucus, and abdominal pain. The course of the disease is characterized by the alternation of acute episodes followed by periods of clinical remission. The medical therapy of this disease is based on administration of anti-inflammatory drugs and immunosuppressants. If not properly treated, chronic inflammation can lead over time to irreversible alterations of intestinal cells with the possible development of cancerous lesions. In rare cases (refractory to medical therapy) it is necessary to make a total colectomy surgery.

The causes of IBD are not yet clear. However, most experts agree that several factors may play a causal role in the disease: genetics and, therefore, familiarity is clearly implicated in the disease; in fact, in 20% of cases, individuals with IBD have a first degree relative (up to first cousins) who suffers from ulcerative colitis or Crohn’s disease; other causes are abnormal reactions of the immune system and, as last, environmental factors. Although the exact cause of IBD is not clear, there are certain triggering factors that can cause a worsening of symptoms.

These include

- stress (in some subjects the emotional stress can lead to an exacerbation of symptoms);
- recently exposure to some types of anti-inflammatory drugs (FANS) or antibiotics;
- intake of some foods;
- smoke.

It is estimated that in Italy about 200,000 people are now suffering from these diseases. The diagnosis of new cases in the last 10 years and the number of patients increased by about 20 times. IBD hit with the same frequency the two sexes, with a clinical onset that is placed between 15 and 45 years. It is important to emphasize that neither the UC or CD are contagious. The two diseases are different, even if they affect the same apparatus. Therefore, a statistical comparison between patients affected by CD and UC is very interesting, from a medical and scientific point of view, in order to assess the differences between them.

## Methods

### Permutation tests: The reasons

Parametric tests usually imply an approach to the hypothesis test problem that require a series of stringent hypotheses, which are often in practice difficult to justify, particularly in medical research [49, 50]. These assumptions are sometimes arbitrarily established. Generally, without any justification, biomedical studies assume:

- multivariate normality;
- random sampling;
- homoschedasticity;
- allocation to treatment is independent.

In other words, the concept that “all models are wrong but some are useful” is often adopted without an adequate critical spirit so that one can be confident that the resulting approximation can be considered acceptable for the specific problem. Conversely, non-parametric statistical tests try to keep assumptions at a lower level, possibly avoiding those that are hard to justify. By doing so, they rely on less stringent and more realistic foundations and are intrinsically robust.

### Permutation tests: The methodology

In this section we introduce the theoretical aspects of Non Parametric Combination (NPC) test, based on permutation solution [36]. Permutation tests [12] represent an effective solution for problems concerning the testing of multiple hypotheses, that are difficult or even impossible to face in a parametric context. This multivariate procedure allows to reach effective solutions concerning problems of multidimensional hypotheses verifying by nonparametric permutation inference [34]; it is used in different application fields that concern verifying of multidimensional hypotheses with a complexity that cannot be managed in parametric context [43].

In comparison to the classical approach, NPC test is characterized by several advantages:

- it does not require normality and homoschedasticity assumptions ([28]; Janssen A. [27];
- it draws any type of variable [35];
- it assumes a good behaviour also in presence of missing data; “without relevant loss of information we may remove from the permutation sample space, associated with the whole data set, all data permutation in which the actual sample size of really observed data are not sufficient for approximation. We must establish a kind of restriction on the permutation space, provided that this restriction does not imply biased effects on inferential conclusion”. The missing data can be missing at random (MAR) or not missing at random (NMAR). “The missing data are missing at random (MAR), if the conditional probability of the observed pattern of missing data given the missing data and the value of the observed data is the same for all possible values of the missing data. If the missing data are missing not at random (MNAR), then in order to make valid parametric inferences, the missing data process must be properly specified. The specification of a model which correctly represents the missing data process seems the only way to eliminate the inferencial bias caused by non-responses in a parametric framework. In the literature, various models have been proposed, most of which concern cases in which non-responses are confined to a single variable.” ([36], pp. 232–243). We can state that the permutation analysis can be run when there is missingness and is valid when we have missing completely at random (MCAR) data. So, NPC test allows to ignore missingness by removing all unobserved units from the data set and to obtain exact permutation solutions;
- it is powerful in presence of low sampling size [9];
- it resolves multivariate problems without the necessity to specify the dependence structure among variables [5, 6, 20];
- it allows stratified analyses;
- it allows to test multivariate restricted alternative hypothesis (to verify the directionality for a specific alternative hypothesis);
- it solves problems in which the number of observed subjects is smaller than that of variables [17].

The NPC method is optimal when you want to identify any different patterns between the layers. It allows to realize the control of possible confounding factors using data post-stratification techniques. For the control of these factors, which is performed by randomization in clinical trials, an observational context is used in the so-called post-stratification. Furthermore, this methodology can also be used with heterogeneous response variables. The NPC method has proven to be robust in the presence of heterogeneity [3].

All these properties make NPC test very flexible [2, 24, 54] and widely applied in several fields; in particular we cite recent applications in medical context [4, 7, 8, 10, 16, 25, 48, 53] and in genetics [14].

By means of the mentioned procedure, it is preliminarily possible to define a set of K one-dimensional permutation test, denominated partial test, through which the marginal contribution of every response-variable can be examined while comparing groups.

The partial tests are non-parametrically combined through CMC (Conditional Monte Carlo) procedure in combined tests, using an opportune combination function (generally Fisher, Tippett or Liptak); these tests globally verify the existence of differences among the multivariate distributions in the groups.

Let us suppose that K variables are observed on two groups (*c = 1,2*)of n_c_ subjects each. So, the observed data are **X** = (X_*icu*_, *i = 1,...,K*; *c = 1,2; u = 1,...,n*_*c*_).

According to Roy’s Union-Intersection notation [45], the null hypothesis states the distributional equality in of two K-dimensional variables, that is

$$ {\mathrm{H}}_0={\mathrm{P}}_1={\mathrm{P}}_2\equiv {\cap}_{i=1}^K\left({\mathrm{X}}_{\mathrm{i}1}\ \overset{d}{=}{\mathrm{X}}_{\mathrm{i}2}\right)={\cap}_{i=1}^K{\mathrm{H}}_{0\mathrm{i}}, $$

where a breakdown into K sub-null hypotheses is emphasized. Indeed, global H_0_ is true if all K sub-null are jointly true. The alternative is.

$$ {\mathrm{H}}_1={\cup}_{i=1}^K\;\left({{\mathrm{X}}_{\mathrm{i}1}}_{\ne}^d\;{\mathrm{X}}_{\mathrm{i}2}\right)={\cup}_{i=1}^K{\mathrm{H}}_{1\mathrm{i}}, $$

which is true when at least one sub-alternative is true.

The distributional equality stated by H_0_ implies that the observed data vectors are exchangeable between two groups. Without loss of generality, we suppose that for each sub-hypothesis H_0i_ against H_1i_ there is a suitable partial permutation test T_i_ assumed to be significant for large values.

The system of hypotheses is set in such a way that the related partial tests are jointly processed, so that they can be combined nonparametrically by taking into account their underlying dependence structure within the nonparametric combination method (NPC). We notice that, especially when the number of variables is large, the underlying dependence structure can be more complex than pair-wise linear, as it is common described by multivariate Gaussian distribution. So, it is impossible to deal with it by proper estimators of all related regression coefficients, the number and type of which are typically unknown. Thus, it must be worked out nonparametrically. This implies turning to the permutation testing principle and specifically to the NPC.

It is worth noting to observe that permutation tests enjoy several important properties. Among these we underline:

- the similarity, that is the rate of rejection of H_0_, when it is true, is α uniformly for all possible sample data and independently whichever the underling distribution;
- under the alternative, the rejection rate of H_0_ is not smaller than α uniformly for all sample data and all underlying distributions, which imply a form of uniform unbiasedness.

The analysis was performed using Methodologica Srl (2001) NPC Test: Statistical Software for Multivariate Permutation Tests (Methodologica Copyright). In the calculation of raw *p*-value 10,000 permutations were implemented.

### The Bonferroni-Holm procedure

The Bonferroni - Holm procedure [26] allows to solve the problem of multiple comparisons [1]; it provides control are the family wise error rate (the probability of witnessing one or more Type I errors), by adjusting the rejection criteria for each hypothesis, and offers a simple method, uniformly more powerful than the classical Bonferroni correction. It works as follows:

1. all *p*-values are sorted from smallest to largest. Let’s indicate with K the number of the *p*-values;
2. if the first *p*-value is greater than or equal to α/K, the procedure is stopped and no p-values are significant. Otherwise, we go on.
3. the first *p*-value is declared significant and afterwards the second p-value is compared to α/(K-1). If the second *p*-value is greater than or equal to α/(K-1), the procedure is stopped and no further p-values are significant. Otherwise, we go on until the *i*-th ordered *p*-value is such that:
*p*_*(i) ≥*_ *α /(K-i + 1).*

Bonferroni-Holm procedure is the most widely recommended way to reduce the apparent significance of effects. The great advantage with the sequentially rejective Bonferroni test (as well as with the classical Bonferroni test) is its flexibility [47]. There are no restrictions on the type of tests, the only requirement being that it should be possible to calculate the obtained level for each separate test.

## Results

In a multicenter retrospective observational study, we investigated the disease occurrence and course in the first three years in 1722 patients followed the Gastroenterology Unit of several Hospitals, located in the Italian territory: Bari, Cagliari, Catania, Desio (Monza and Brianza), Florence, Messina, Milan, Naples, Padua, Palermo, Rome, San Giovanni Rotondo (Foggia).

The data, deriving from the various hospital centers, were organized in a single dataset by Prof. Walter Fries, Director of Gastroenterology Unit of the University Hospital “G. Martino” in Messina (see [21, 22]). The distribution of outcomes does not vary by center because we verified the condition of equality among the means of the covariates in the different centers, applying the Analysis of Variance (ANOVA) test; it provided non-significant results for all variables, denoting the existence of similarities among the means. Before applying the NPC test methodology we also assessed possible heterogeneity or homogeneity in the data, deriving from the different centers through the application of Levene’s test; it was used to assess if 12 samples, deriving from the twelve hospital centers had equal variances. Since the test was not significant for all the examined variables, the condition of “homogeneity of variances” in the data coming from the different centers was established.The analysis was performed in order to assess the existence of significant differences between patients affected by CD and UC, in the context of the IBD. Specifically, we examined data concerning 631 CD patients (36.6%) and 1091 UC patients (63.4%). Disease patterns, medical and surgical therapies, and risk factors for disease outcomes were analyzed. In particular, for each patient (in the respect of privacy) we acquired information about twenty-two variables: diagnosis age, gender, smoking habit (yes or no), use of immunosuppressive therapy (yes or no) and its duration, treatment with biological drugs (yes or no) and its duration, re-hospitalization (yes or no), adverse events (yes or no), infections (yes or no), cancers (yes or no), diabetes (yes or no), hypertension (yes or no), heart failure (yes or no), kidney failure (yes or no), pulmonary failure (yes or no), neuropathy (yes or no), liver disease (yes or no), Charlson Index (the most widely used index to predict the ten-year mortality for a patient who may have comorbidity conditions; its score are 1, 2, 3 or 6, depending on the risk of death), surgery (yes or no), final exitus (survivor or died) and follow-up time. The hypotheses system is the following:

$$ {\mathrm{H}}_0:\left\{\mathrm{diagn}.{{\mathrm{age}}_1}_{=}^d\;\mathrm{diagn}.{\mathrm{age}}_2\right\}\cap \dots \cap \left\{\mathrm{foll}.{{\mathrm{time}}_1}_{=}^d\;\mathrm{foll}.{\mathrm{time}}_2\right\} $$

$$ {\mathrm{H}}_1:\left\{\mathrm{diagn}.{{\mathrm{age}}_1}_{\ne}^d\;\mathrm{diagn}.{\mathrm{age}}_2\right\}\cup \dots \cup \left\{\mathrm{foll}.{{\mathrm{time}}_1}_{\ne}^d\;\mathrm{foll}.{\mathrm{time}}_2\right\} $$

where 1 and 2 are the two examined inflammatory bowel diseases.

We used “Likelihood Ratio test” for categorical variables and “differences for two means test” for numerical ones. The used statistical package was NPC test, version 2.0, Statistical Software for Multivariate Nonparametric Permutation Test, Copyright 2001, Methodologica s.r.l.

In Table 1 we report, for both groups of patients, mean ± standard deviations (for numerical variables) and percentages (for categorical variables). The last column of the Table 1 shows the partial *p*-values obtained by the application of NPC Test for analyzing the differences between the two examined groups; the last row shows the combined p-value, referred to all twenty-two variables.

**Table 1:** NPC test for comparisons between CD and UC patients

Variables	Crohn’s disease	Ulcerative colitis	*p*-value
Diagnosis age	43.80 ± 21.59	45.87 ± 20.83	*0.0281*
Gender (M% /F%)	49.3 /50.7	57.7/42.3	*0.0019*
Smoking habit	43.9 /56.1	35.2 / 64.8	*0.0001*
Immun. therapy	38.4/61.6	26.4 / 73.6	*0.0002*
Duration of therapy	12.27 ± 8.67	14.92 ± 9.84	*0.0032*
Biological drugs (BD)	18.4/81.6	8.7/91.3	*0.0004*
Duration of BD	12.23 ± 7.33	16.03 ± 9.41	*0.0053*
Re-hospitalization	45.6 / 54.4	27.8 / 72.2	*0.0008*
Adverse events	17.9 / 82.1	9.2 / 90.8	*0.0007*
Infections	7.0/93.0	7.6 / 92.4	0.6274
Cancers	3.6 / 96.4	2.5 / 97.5	0.2755
Diabetes	5.6 / 94.4	10.6/89.4	*0.0006*
Hypertension	20.3 / 79.7	19.6/80.4	0.7376
Heart failure	5.7 / 94.3	5.7 / 94.3	0.9852
Kidney failure	2.1 /97.9	2.6 / 97.4	0.5074
Pulmonary failure	3.6/96.4	5.1 /94.9	0.1553
Neuropathy	1.7/98.3	1.6/98.4	0.8858
Liver disease	1.6/98.4	1.3 / 98.7	0.6075
Charlson score	1.47 ± 1.81	1.20 ± 1.39	*0.0316*
Surgery	19.7 /80.3	5.3 / 94.7	*0.0003*
Final exitus	97.9/2.1	97.6 / 2.4	0.6643
Follow-up time	34.57 ± 4.54	34.92 ± 4.16	0.0577
COMBINED *p*-value			*0.0000*

Note: the values in italics identify the significant variables (level α = 0.05)

 Examining the results achieved by applying NPC tests for partial and combined tests, we have to notice the high significance of the combined test, that provides guarantee affirming that patients with Crohn’s Disease and Ulcerative Colitis significantly differ between them, in relation to the examined variables. Focusing our attention on raw *p*-values of partial tests, we can see that some variables significantly discriminate the two different subpopulations; in particular the UC patients, in compared to the CD patients, have a higher diagnosis age, do not show a marked smoking status, the proportion of patients treated with immunosuppressants or with biological drugs is lower than the CD patients, even if the duration of such therapies is longer. CD patients have a higher rate of re-hospitalization; probably this is related to the significant greater occurrence of adverse events (rather than UC). Diabetes is more present in the sub-population of UC patients. Analyzing the Charlson score we can highlight that UC patients have a more severe clinical situation than CD patients. Finally, the CD patients are more frequently subject to surgery compared to UC.

Since we are in presence of a high number of variables, we applied the Sequentially Rejective Multiple Test to determine which of the partial tests are effectively significant into discrimination between CD and UC patients.

In Table 2 we report, for each variable, the raw *p*-values, the *i-*index (number expressing the ascending sort of raw *p*-values) and the adjusted *p*-values. Examining the raw p-value (obtained from the NPC test), we note that twelve variables are apparently significant. After application of the Bonferroni Holm procedure, which provided adjusted p-values, we can note that only nine of these variables were statistically significant; in accordance to *i*-index, they are: Smoking habit, Immunosuppressive therapy, Surgery, Biological Drug, Diabetes, Adverse Events, Re-hospitalization, Gender and Duration of Immunosoppressive Therapy. So, with our data we can conclude that they are the only variables that significantly discriminate the Crohn’s Disease and Ulcerative Colitis groups.

**Table 2:** Application of sequentially rejective multiple test procedure

Variables	Raw *p*-value	i	Adj. *p*-value
Diagnosis age	0.0281	11	0.3372
Gender	0.0019	8	0.0285
Smoking habit	0.0001	1	0.0022
Immunosuppressive therapy	0.0002	2	0.0042
Duration of immunosuppr. Therapy	0.0032	9	0.0448
Biological drugs	0.0004	4	0.0076
Duration of biological treatment	0.0053	10	0.0689
Re-hospitalization	0.0008	7	0.0128
Adverse events	0.0007	6	0.0119
Infections	0.6274	18	0.9999
Cancers	0.2755	15	0.9999
Diabetes	0.0006	5	0.0108
Hypertension	0.7376	20	0.9999
Heart failure	0.9852	22	0.9999
Kidney failure	0.5074	16	0.9999
Pulmonary failure	0.1553	14	0.9999
Neuropathy	0.8858	21	0.9999
Liver disease	0.6075	17	0.9999
Charlson score	0.0316	12	0.3476
Surgery	0.0003	3	0.0060
Final exitus	0.6643	19	0.9999
Follow-up time	0.0567	13	0.5770

## Discussion

In general, IBDs affect 2.2 million people in Europe [15] and in Italy the estimated incidence of ulcerative colitis is 5.2 cases per 100,000 inhabitants per year, with a prevalence of approximately 70,150 cases / 100,000, and for Crohn’s disease 2.3 cases per 100,000 inhabitants per year, with a prevalence of 20–40 cases / 100,000 [42, 51].

In particular, we know that Crohn’s disease is spread all over the world and reaches the highest prevalence in Western nations. The ratio of affected females and males is around 1.35: 1 and many studies show that smokers are twice as likely to develop Crohn’s disease compared to non-smokers [11, 13]. Our study, in line with previous literature, shows how CD patients, when compared with UC patients, do not exhibit a marked “smoker status” in the sense that smoking is more a cause of Crohn’s disease than of Ulcerative Colitis. Avoiding smoking, in a way, helps reduce the likelihood of contracting the disease.

IBDs can lead to various complications within the intestine, including obstruction, fistula and abscess development, as well as increase the risk of cancer in the inflammation area. For example, individuals with Crohn’s disease involving the small intestine are at greater risk for intestinal cancer. There is no certainty care yet [52].

Unfortunately, the IBD cannot be promptly prevented [29], even if complications and evolution can be prevented. Our analysis has made it possible to show more accurately the variables that most cause this disease. For this reason it is recommended to focus on the latter for prevention purposes.

As recalled several times, the use of non-parametric tests makes it possible to narrow the range of significant variables to focus on those of the most critical for preventive purposes.Ultimately, our analysis has made it possible to outline the variables that most discriminate these diseases.

From the statistical point of view, in this paper, one of the purposes was to examine and critically discuss the theoretical and practical relevance of permutation tests, demonstrating their effectiveness and ease of use in medical research. In literature NPC permutation tests have been successfully applied in many bio-medical and epidemiologic fields, including gastroenterology [18, 19].

For statistical properties, the permutation tests have interesting property; in particular they are exact for any, even very small, sample size. This means that their null distributions, which are used to compute the *p*-value, are known for each data set and for each sample size and this implies to controlling I and II error types. On the contrary, non-parametric tests are asymptotically guaranteed only for large sample sizes.

Besides, considering simultaneously different hypotheses, the problem of multiplicity or multiple testing problem arises. An incorrect approach is to test each hypothesis separately, using some level of significance α; in this case the real α level is bigger than nominal fixed level. Besides, the multiple testing approach consist to test simultaneous the set of hypoteses null and to use some appropriate correction to reached the desidered α level.

Specifically, the Holm-Bonferroni method is an approach that controls the probability that one or more type I errors will be adjusted, using adequate criteria for rejecting each of the individual hypotheses or comparisons. The comparison between groups is complex for the presence of multiple variables. This problem with parametric methods cannot be solved because the assumptions are too stringent.

NPC tests outweigh some of the limitations that traditional multivariate hypothesis verification procedures have, such as the ability to include a large number of variables. At the same time NPC tests offer a large number of advantages:

- this is an exact inferential procedure for any finite size of the sample;
- the solution is robust compared to the actual random distribution below the data (or error);
- the NPC procedure implicitly takes into account the underlying dependency structure of the response variables;
- it is not affected by the problem of loss of degrees of freedom when the number of variables increases.

Indeed, in contrast to traditional methods, increasing the number of information outputs also the power of the NPC test increases, i.e. the probability of detecting a true effect also increases monotonously [37]. In this sense, the NPC methodology can provide an effective and robust tool for statistical analysis of both experimental and observational medical studies.

In particular, in this paper we tried to show as the permutation tests are helpful for large-sized data analysis in many applications contexts. In large data sets consisting of 1000 or more observations, performance of the permutation test appears equivalent to that of the asymptotic test; on the other hand, the NPC test, based on permutation solution, can be appropriately applied when the assumption for asymptotic tests are fulfilled [30]. In addition, unlike the classical nonparametric tests, the NPC method entails testing a global null hypothesis consisting of the intersection of K > 1 partial sub-hypotheses. In essence, the global null states that all of its constituent sub-hypotheses are true. \par The global alternative hypothesis is the union of K sub-alternatives. In this way NPC provides in multivariate context the combined *p*-value, by means of an adequate combining function.

From the application point of view, we have great interest in evaluating this combined p-value because it provides a result that takes into account the contribution of all examined variables; on the other hand, no other non-parametric test provides the advantage of a combined *p*-value [41].

This particular feature justifies our choice of the NPC test as methodologically appropriate solution. In particular we applied permutation tests to perform comparison between a large number of patients affected by Crohn’s Disease and Ulcerative Colitis. Both of these illness are inflammatory bowel diseases, involving more than 100,000 people in Italy; they often arise in young people, go on for a lifetime and manifest alterations of the intestinal canal, causing relationship and working problems.

The results achieved applying NPC tests underline the high significance of the combined test, that shows that patients with Crohn’s Disease significantly differ from Ulcerative Colitis patients. Looking at the partial tests, we can notice that the differences between groups are referable to most of the examined variables; in particular the UC patients have a higher diagnosis age than CD patients, not showing a marked smoking status, the proportion of patients treated with immunosuppressants or with biological drugs is lower than the CD patients, even if the duration of these therapies is longer. On the other hand, CD patients have a higher rate of re-hospitalization; probably it is related to the significant greater occurrence of adverse events (rather than UC). Diabetes is more recurrent in UC group. Moreover, UC patients have a more severe clinical profile, such as defined by Charlson score. Finally, the CD patients are more frequently subjected to surgery.

The findings of the study have a limit, which is represented by the sampling plan. Since the patients followed in the different hospital centers were examined and enrolled in the analyzed sample, we must admit that a sampling of convenience was chosen; it provides for the selection of the sample on the basis of criteria of convenience or practicality; it does not offer to all units of the population the same possibility of becoming part of the sample.

## Conclusions

From a methodological point of view, thanks to Bonferroni-Holm procedure we were able to identify the really significant variables that discriminate the groups in exam, satisfying the multiplicity problem [40]. On the bases of the results we can affirm that Smoking habit, Immunosuppressive therapy, Surgery, Biological Drug, Diabetes, Adverse Events, Re-hospitalization, Gender and Duration of Immunosoppressive Therapy are the variables effectively significant.

It is notable that the Bonferroni-Holm procedure leaves unchanged the original data information and allows a better interpretation of the results.

Until a few years ago the use of large-sized data did not receive particular attention from researchers. Today the conspicuous availability of large amounts of data and the need of their analysis requires an adjustment of data processing methodologies, with careful attention to all the sources of variation in data. In this context, the non-parametric procedures, such as permutation tests, are widely applicable because of the numerous optimal properties of which they are characterized.

In the end, we can argue that the causes of IBD are not yet clear. In this paper we have identified the really significant variables that discriminate the groups under exam, satisfying the multiplicity problem. In fact we can affirm that Smoking habit, Immunosuppressive therapy, Surgery, Biological Drug, Diabetes, Adverse Events, Re-hospitalization, Gender and Duration of Immunosuppressive Therapy are the effectively significant variables which can explain the occurrence of these diseases.

This work does not intend to provide a contribution in the clinical field of the IBD literature, but wants to allow a reflection on the possibility of using the NPC methodology to compare two chronic diseases (CD and UC) that affect the intestine (just the IBD) but which differ in some specific aspects.

It seems that the incidence of CD affects males and females with the same frequency (even if several studies allow to affirm that the female sex, especially if under the age of 45, presents a 20–30% greater risk of Crohn’s disease compared to males).

Among the environmental factors, the most important is the smoke that, curiously, seems to predispose to the CD rather than to the UC. IBDs are diseases that require medical therapy, close clinical surveillance and an appropriate therapeutic regimen. Medical therapy is based on the use of drugs such as immunosuppressant and biological drugs, but patients with CD are more frequently being subjected to such forms of therapy.

Medical therapy aims to induce clinical remission of the disease and keep patients free from relapses of the disease. In fact, the statistical comparison reveals that patients with CD (rather than UC) more frequently report cases of hospitalization due to IBD; probably it is related to the significant greater occurrence of adverse events.

Diabetes is more recurrent in UC group; this is already known because type 1 diabetes is the third most common co-morbidity in patients with UC (after psoriasis and rheumatoid arthritis). However, diabetes can also complicate post-operative recovery in patients suffering from ulcerative colitis.

Moreover, UC patients have a more severe clinical profile, such as defined by Charlson score, the co-morbidity of ulcerative colitis with other disorders of an EXTRA-intestinal nature is very frequent.

Finally, the CD patients are more frequently subjected to surgery: surgery is an almost obligatory stage in the natural history of Crohn’s disease (about 70% of cases). The surgical intervention of intestinal resection, however, is almost invariably followed by recurrence of lesions (endoscopic relapse) and symptoms (clinical relapse).

## Abbreviations

ANOVA: Analysis of variance
CD: Crohn’s disease
IBD: Inflammatory bowel diseases
MAR: Missing at random
MCAR: Missing completely at random
NMAR: Not missing at random
NPC: Non-Parametric combination
UC: Ulcerative colitis
